# Power Quality Improvement by Unified Power Quality Conditioner Based on CSC Topology Using Synchronous Reference Frame Theory

**DOI:** 10.1155/2014/391975

**Published:** 2014-06-11

**Authors:** Rajasekaran Dharmalingam, Subhransu Sekhar Dash, Karthikrajan Senthilnathan, Arun Bhaskar Mayilvaganan, Subramani Chinnamuthu

**Affiliations:** ^1^Department of Electrical and Electronics Engineering, RMD Engineering College, Chennai, India; ^2^Department of Electrical and Electronics Engineering, SRM University, Chennai, India; ^3^Department of Electrical and Electronics Engineering, Velammal Engineering College, Chennai, India

## Abstract

This paper deals with the performance of unified power quality conditioner (UPQC) based on current source converter (CSC) topology. UPQC is used to mitigate the power quality problems like harmonics and sag. The shunt and series active filter performs the simultaneous elimination of current and voltage problems. The power fed is linked through common DC link and maintains constant real power exchange. The DC link is connected through the reactor. The real power supply is given by the photovoltaic system for the compensation of power quality problems. The reference current and voltage generation for shunt and series converter is based on phase locked loop and synchronous reference frame theory. The proposed UPQC-CSC design has superior performance for mitigating the power quality problems.

## 1. Introduction

The main impact in the power distribution system is the quality of power, which causes more distortion in the source due to using nonlinear loads (power electronics loads). The main cause for distortion is harmonics, notching, and interharmonics. Distortion is that the fundamental frequency sine wave is represented as super position of all harmonic frequency sine waves on fundamental sine wave. The usage of power electronics loads is increased day by day, while considering that industries power electronics drives are used for the automation of the industries. To compensate the distortion in the system, passive filters were used and while using the passive filters particular harmonic range is only eliminated. In order to overcome the drawbacks of passive filter, for the elimination of power quality problems, active filters were used.

Power quality problems are harmonics, sag, and swell which are mitigated by the active filters by the configuration of dynamic voltage restorer (DVR), distribution-static synchronous compensator (D-STATCOM), and unified power quality conditioner (UPQC) [1]. In this paper UPQC [2] is used for the mitigation of the power quality problems which is the combination of series and shunt active filters. The series and shunt active power filters are voltage and current source converters which are controlled by the PWM signals which are generated by the controllers.

## 2. Unified Power Quality Conditioner (UPQC)

The unified power quality conditioner is commonly called UPQC. The design configuration is based on the connection of series and shunt inverters. In this, the design configuration is right series and left shunt with the current source converter (CSC) [3, 4]. In this paper, UPQC-CSC [5, 6] is designed and analysis of the results has been done. Unified power quality conditioner (UPQC) for nonlinear and voltage sensitive load has following facilities.

- It reduces the harmonics in the supply current, so that it can improve utility current quality for nonlinear loads.
- UPQC provides the VAR requirement of the load, so that the supply voltage and current are always in phase; therefore, no additional power factor correction equipment is required.
- UPQC maintains load end voltage at the rated value even in the presence of supply voltage sag.

**Figure 1:**
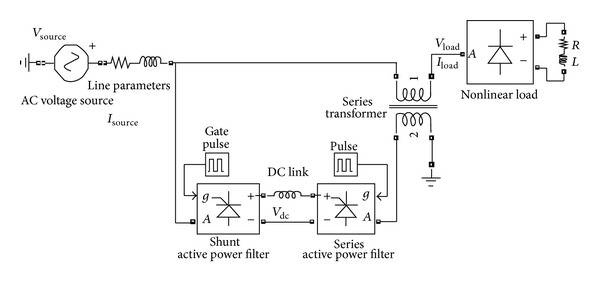
The design configuration of UPQC-CSC.

The design configuration of UPQC-CSC [7] is shown in Figure 1.

## 3. Synchronous Reference Frame (SRF) Theory

The control strategy for the unified power quality conditioner is based on the synchronous reference frame (SRF) [8, 9] theory. In this theory controlling of the three-phase converters using the rotating frame theory by converting the source voltage and current to direct and quadrature axis is done. The voltage is converted to *dq* in the series controller and current is converted to *dq* in the series controller. Consider

(1) [VaVbVC] =23[121212sin(wt)sin(wt−2π3)sin(wt+2π3)cos⁡(wt)cos⁡(wt−2π3)cos⁡(wt+2π3)][VdVqV0].

The *dq* transform is again converted to the *V*_*abc*_′ in order to get the reference signal which is used for the generation of the pulse for the three-phase converter in the system. Consider

(2) $$\begin{array}{c} \left[\begin{array}{c} V_{d}\\ V_{q}\\ V_{0} \end{array}\right]=\sqrt{\frac{2}{3}}\left[\begin{array}{ccc} \frac{1}{\sqrt{2}} & \sin\left(\text{wt}\right) & \cos\left(\text{wt}\right)\\ \frac{1}{\sqrt{2}} & \sin\left(\text{wt}-\frac{2\pi }{3}\right) & \cos\left(\text{wt}-\frac{2\pi }{3}\right)\\ \frac{1}{\sqrt{2}} & \sin\left(\text{wt}+\frac{2\pi }{3}\right) & \cos\left(\text{wt}+\frac{2\pi }{3}\right) \end{array}\right]\left[\begin{array}{c} V_{a}^{\prime }\\ V_{b}^{\prime }\\ V_{C}^{\prime } \end{array}\right]. \end{array}$$

**Figure 2:**
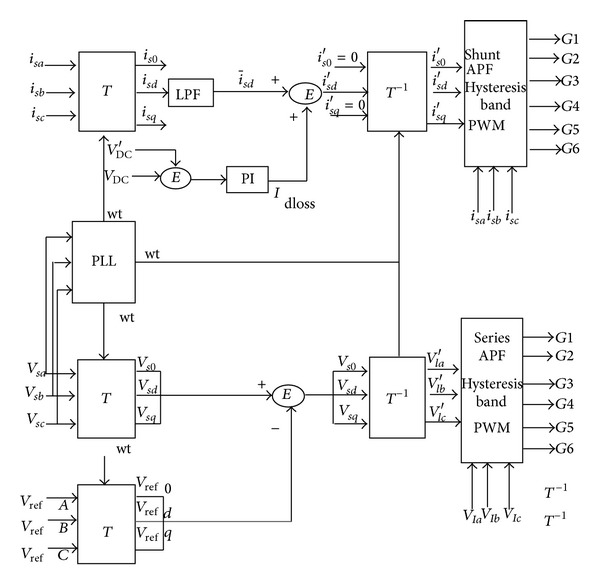
Control block diagram.

The shunt converter performs the process of elimination of harmonics and series converter performs process of elimination of the voltage related problems. The control block diagram for the synchronous reference frame theory is shown in Figure 2.

### 3.1. Series Controller

**Figure 3:**
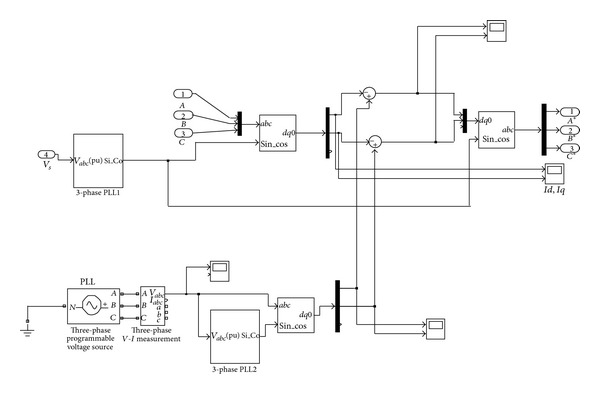
Simulation of synchronous reference frame theory based series controller.

The control strategy of the series controller is achieved through the synchronous reference frame theory. In this, the series controller gets the reference signal for the generation of pulse for the three-phase converter, by comparing the source voltage with distortion and constant voltage. The source voltage *V*_*s* *abc*_ and constant voltage *V*_ref *abc*_ are converted to the *V*_*s* *dq*0_ and *V*_ref *dq*0_ transform. The *V*_*s* *dq*0_ and *V*_ref *dq*0_ are compared to get the error signal which is again converted to *V*_*labc*_′. The *V*_*labc*_′ is the reference signal for the pulse generator. The simulation diagram for synchronous reference frame theory based series controller is shown in Figure 3.

### 3.2. Shunt Controller

**Figure 4:**
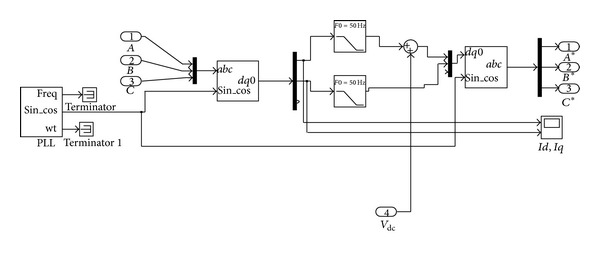
Simulation of shunt controller.

The shunt converter has the function of compensating the current related problems. Along with the shunt controller, DC link voltage is maintained. The *abc* to *dq*0 transform is inversed and converted to *abc*; that signal is given as the reference signal and the measured signal is given to the hysteresis band PWM to produce the pulse signals for the operation of shunt converter. The simulation diagram for shunt controller is shown in Figure 4.

### 3.3. DC Link Controller

**Figure 5:**
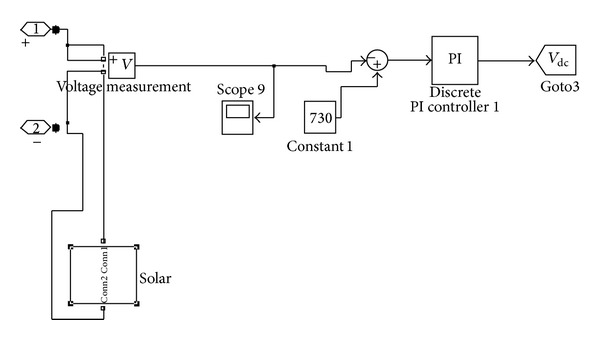
DC link controller.

The direct current link controller has the PI controller in which the constant voltage is given as the set point and the measured voltage is given for the comparison to maintain the constant voltage. The PV array is attached with the DC link for injection. The DC link controller is shown in Figure 5.

## 4. Simulation and Results

**Figure 6:**
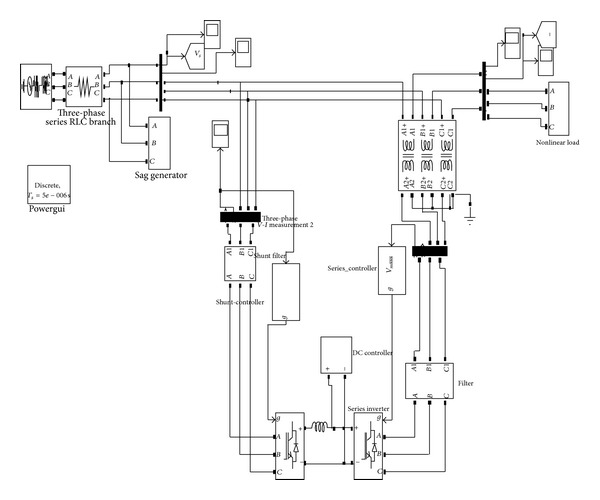
UPQC-CSC simulation diagram.

**Figure 7:**
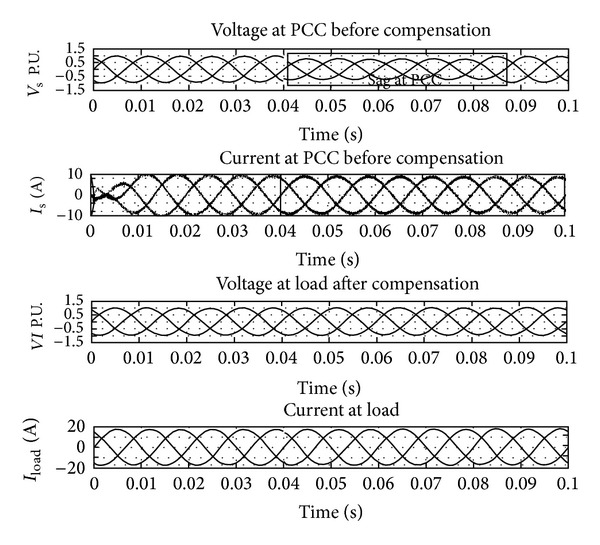
Output of source voltage and current and load voltage and current waveform.

**Figure 8:**
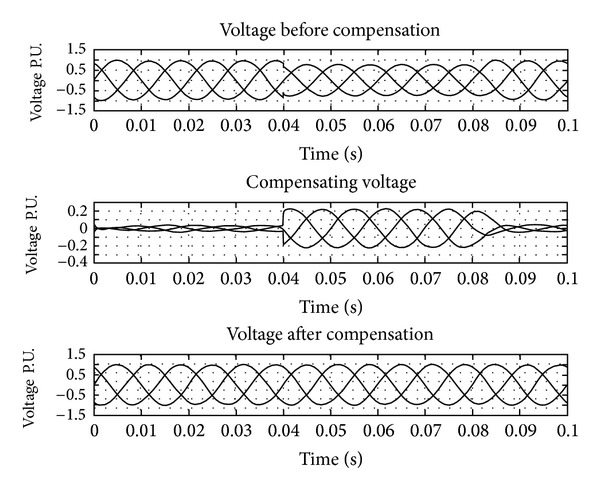
PCC voltage with sag, compensating voltage and voltage after compensation.

**Figure 9:**
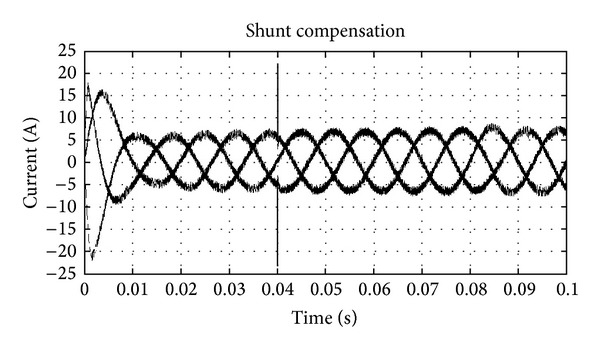
Shunt injection for THD compensation.

**Figure 10:**
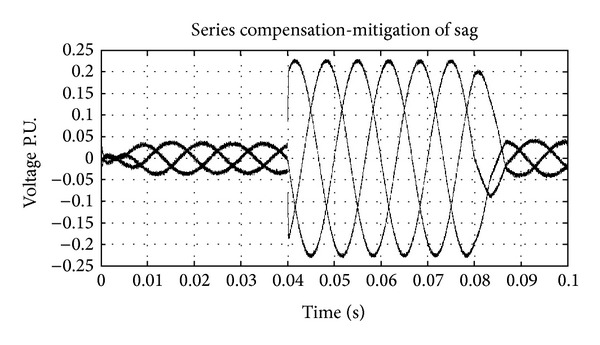
Series injection for sag compensation.

The UPQC-CSC has the reactor as the DC link for the series and shunt converter and is controlled by the synchronous reference frame (SRF) theory and the pulse is generated by the hysteresis band controller. The shunt and series converters have the function of compensating current and voltage problems, respectively. The simulation of UPQC-CSC is shown in Figure 6. The output of UPQC-CSC is shown in Figure 7 which shows the voltage with sag, current with harmonics, and compensated voltage and current. The compensation of sag is shown in Figure 8. The shunt compensation is shown in Figure 9. The series compensation is shown in Figure 10.

### 4.1. System Parameters

Consider

 source voltage: 415 V, 50 Hz;
 load parameters:
   resistive load: 10 KΩ;
   inductive load: 2 mH;
   RLC load: 10 KW;
 shunt inverter side:
   LC filter: 3.5 mH, 5 Ω, and 10 *μ*F;
 series inverter side:
   LC filter: 12 *μ*H, 5 Ω, and 10 *μ*F;
 DC link reactor:
   for UPQC-CSC: 200 mH;
   solar voltage: 727.1 V.

Figure 7 shows the simulation output of the UPQC-CSC simulation for voltage sag mitigation. The sudden addition of load in the system causes voltage sag for the time duration of 0.04 to 0.08 s. The compensation for the sag is by the series active filter using the SRF theory for the reference signal generated and pulse generated by the hysteresis band and given to the IGBTs in the filter.

The compensation of the voltage related problems is done by the series active filter to maintain the system voltage 1 P.U. By using the SRF theory even a minor disturbance in the system is sensed and compensation is done; Figure 10 shows the series compensation for the system. The harmonics compensation is done by the shunt active filter along with the DC link voltage controller. Total harmonics distortion (THD) for the current source converter is shown in Table 1. Figure 9 shows the compensation given for reducing the harmonics.

**Figure 11:**
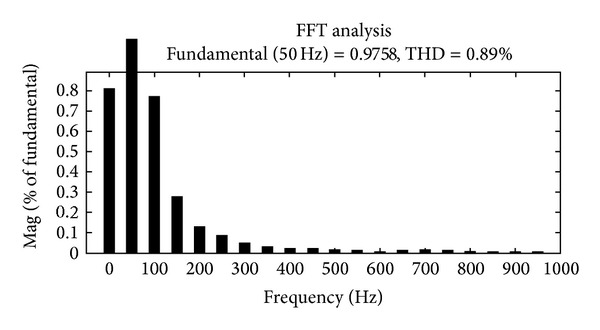
Source voltage THD graph.

The Fourier fast transform analysis graph for the source voltage THD of about 0.89% is shown in Figure 11.

**Figure 12:**
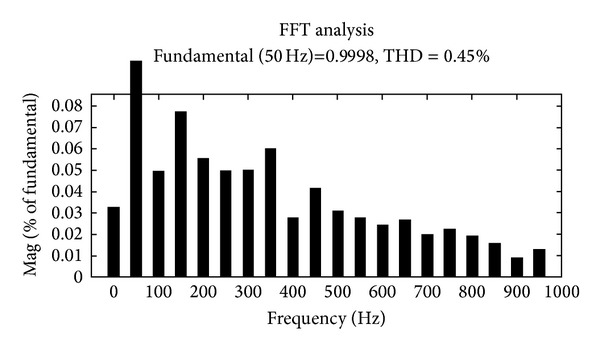
Load voltage THD graph.

The Fourier fast transform analysis graph for the load voltage with the nonlinear loading conditions of about 0.45% is shown in Figure 12.

**Figure 13:**
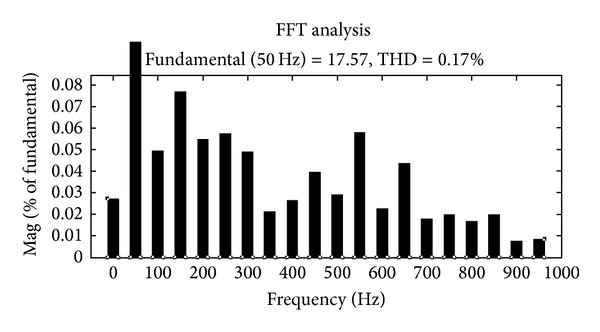
Load current THD graph.

The Fourier fast transform analysis graph for the load current with the nonlinear loading conditions of about 0.17% is shown in Figure 13.

## 5. Conclusion

In this paper, synchronous reference frame theory based control method is implemented to control the working of unified power quality conditioner based on current source converter topology. The simulation results show that the device is capable of compensating the current harmonics under unbalanced and nonlinear load conditions, simultaneously mitigating voltage sag and swell. The proposed UPQC-CSC design has superior performance for mitigating the power quality problems. The series converter is capable of mitigating the voltage related problems and shunt converter is capable of mitigating the harmonics.

## Figures and Tables

**Table 1:** Total harmonics distortion (THD in %).

Current source converter			
H*n* order	Source voltage (*V*_*s*_) in %	Load voltage (*V*_*L*_) in %	Load current (*I*_*L*_) in %
H	0.97	0.99	0.04
H3	0.28	0.08	0.08
H5	0.09	0.05	0.06
H7	0.03	0.06	0.02
H9	0.03	0.04	0.04
H11	0.02	0.03	0.06
THD	0.89	0.45	0.17
